# Androgens and Parkinson's Disease: A Review of Human Studies and Animal Models

**DOI:** 10.1089/andro.2021.0011

**Published:** 2021-12-23

**Authors:** Mélanie Bourque, Denis Soulet, Thérèse Di Paolo

**Affiliations:** ^1^ Centre de Recherche du CHU de Québec-Université Laval, Axe Neurosciences, Québec, Canada.; ^2^ Faculté de pharmacie, Pavillon Ferdinand-Vandry, Université Laval, Québec, Canada.

**Keywords:** Parkinson's disease, androgen, testosterone, dihydrotestosterone, sex differences

## Abstract

Parkinson's disease (PD) is the second most common neurodegenerative disorder after Alzheimer's disease. A greater prevalence and incidence of PD are reported in men than in women, suggesting a potential contribution of sex, genetic difference and/or sex hormones. This review presents an overview of epidemiological and clinical studies investigating sex differences in the incidence and symptoms of PD. This sex difference is replicated in animal models of PD showing an important neuroprotective role of sex steroids. Therefore, although gender and genetic factors likely contribute to the sex difference in PD, focus here will be on sex hormones because of their neuroprotective role. Androgens receive less attention than estrogen. It is well known that endogenous androgens are more abundant in healthy men than in women and decrease with aging; lower levels are reported in PD men than in healthy male subjects. Drug treatments with androgens, androgen precursors, antiandrogens, and drugs modifying androgen metabolism are available to treat various endocrine conditions, thus having translational value for PD but none have yet given sufficient positive effects for PD. Variability in the androgen receptor is reported in humans and is an additional factor in the response to androgens. In animal models of PD used to study neuroprotective activity, the androgens testosterone and dihydrotestosterone have given inconsistent results. 5α-Reductase inhibitors have shown neuroprotective activity in animal models of PD and antidyskinetic activity. Hence, androgens have not consistently shown beneficial or deleterious effects in PD but numerous androgen-related drugs are available that could be repurposed for PD.

## Introduction

Parkinson's disease (PD) is a chronic progressive neurodegenerative disease, with clinical manifestations resulting from gradual but extensive loss of dopamine (DA) neurons in the brain substantia nigra pars compacta.^1^ Except for familial cases, PD is rarely observed before age 50 years but its prevalence is about 1% in people over 60 years of age.^2^ The appearance of rigidity, bradykinesia, postural instability, and resting tremor are the clinical hallmarks of PD.^1^ Nonmotor symptoms are also present including a variety of cognitive, neuropsychiatric, sleep, autonomic, and sensory disturbances.^3^

PD can be linked to gene mutations in familial forms but the etiology of the majority of PD cases is currently unknown and most likely involves the interaction of genetic, epigenetic, and environmental risk factors.^4^

Sex differences in PD have been reported in epidemiological and clinical studies as well as in response to treatments.^5^ Epidemiological studies have documented that both the incidence and prevalence of PD are higher in men than in women, men are at least 1.5 times more likely to develop PD than women.^6–13^ The age of onset of PD appears about 2 years earlier in men.^14,15^ A longer reproductive lifespan is associated with a delay in age of onset,^14,16,17^ suggesting that longer exposure to circulating endogenous estrogen levels throughout a woman's life has a positive effect and that estrogens can act as a protective agent. The epidemiological evidence of sex differences in PD suggests a possible beneficial activity of female gonadal hormones, and this aspect has been extensively reviewed.^5,18,19^ Androgens have received less attention with respect to their potential effects in PD, and their implication will be reviewed here.

## Endogenous Androgens and PD

Testosterone is one of the main androgenic steroids synthesized by the testis. Testosterone is biotransformed to dihydrotestosterone by 5α-reductase enzymes, or into estradiol by an aromatization process.^20^ Androgens' action is mediated by binding to classical androgen receptor or membrane androgen receptor.^21,22^ The variability in the androgen receptor reported in human confers differences in receptor function and then distinct response to androgens.^23,24^

During normal aging, levels of testosterone in men slowly decrease in a progressive rate from the fourth or fifth decade.^25^ Incidence of testosterone deficiency increased to 12%, 19%, 28%, and 49% for men over ages 50, 60, 70, and 80 years, respectively.^26^ Since the incidence of PD is higher in men than in women and ovarian hormones could be a protective factor in women, an important question is to determine whether there is an association between androgen levels and PD in men (Table 1). Two studies,^27,28^ each with a small number of PD patients, suggested a higher prevalence of low testosterone in these patients than during normal aging,^26^ but this has to be confirmed in larger studies. In addition to androgen contents, androgen receptor levels could play a role in the effect of androgens in PD. However, the mRNA levels of the androgen receptor in substantia nigra were reported not different between male and female PD patients and matched those of controls.^29^ As testosterone can be synthesized in the brain, plasma testosterone levels do not necessarily reflect the levels found in the brain. To our knowledge, measures of testosterone levels in the brain of PD patients have not been reported.

**Table 1.** Testosterone and Clinical Studies in Parkinson's Disease

Endogenous androgen and PD			
Sample description	Main results		Refs.
PD and testosterone levels			
68 patients with PD	The prevalence of low testosterone levels in PD patient was 35%.		^27^
50 of the 91 patients with PD were screened with free testosterone levels.	Half the PD patients who were screened were defined as having low testosterone levels.		^28^
Reduction of testosterone levels and incidence of PD			
1335 patients with prostate cancer compared with 4005 age-matched patients.	Androgen deprivation therapy in patients with prostate cancer was not associated with a higher risk of PD.		^31^
38,931 patients with prostate cancer on continuous androgen deprivation therapy and 34,272 matched patients.	Androgen deprivation therapy in patients with prostate cancer was associated with a lower risk of PD.		^32^

**Androgen treatment on PD symptoms**			
**Treatment**	**Study description**	**Main results**	**Refs.**
Testosterone			
A single daily dose of testosterone topical gel (5 g/day of Androgel [equivalent of 5 mg/day of testosterone]) for 1 month. Six of the 10 patients were also followed up for 3 months.	A prospective open-labeled pilot study in 10 testosterone-deficient men with PD.	No effect on the UPDRS parts II and III, the Obeso dyskinesias rating scale. The UPDRS part IV improved at 1 month but not at 3 months. The UPDRS part I improved at the 3-month follow-up visit.	^46^
Intramuscular testosterone esters 100 mg monthly for 3 months and increasing to 250 mg monthly.	A case report of an 80-year-old man with PD with testosterone deficiency.	Improvement in resting tremor and fine motor control after testosterone administration correlated with serum testosterone levels.	^47^
200 mg/mL of testosterone enanthate every 2 weeks for 8 weeks.	A double-blind, placebo-controlled trial (15 PD patients in the placebo group, 15 PD patients in the testosterone group).	No effect on the UPDRS scale.	^45^
A single daily dose of testosterone topical gel (5 g/day of androgel (equivalent of 5 mg/day of testosterone)).	A retrospective analysis of five patients with combined PD and symptom of testosterone deficiency.	Several PD patients described an improvement in their PD symptoms, but this was not always associated by a change in the UPDRS motor score.	^27^
5α-Reductase inhibitor			
Finasteride 5 mg/day	Case reports of two PD patients with pathological gambling.	Finasteride attenuated pathological gambling symptoms of PD patients.	^70^
Androgen receptor inhibitor			
Spironolactone 100 mg/day	A case report of a 72-year-old man with PD and congestive heart failure.	Worsening of the ON state UPDRS part III. After withdrawal of spironolactone, motor function returns to baseline values.	^59^

PD, Parkinson's disease; UPDRS, Unified Parkinson Disease Rating Scale; Part I, nonmotor experiences of daily living; Part II, motor experiences of daily living; Part III, motor examination; Part IV, motor complications.

Another aspect to consider that could potentially influence testosterone levels is dopaminergic treatments. Levodopa, the precursor of DA (gold standard treatment for PD), or the DA receptor agonist pramipexole treatments in early PD, do not reduce testosterone levels but rather have been reported to slightly increase them, thus the decrease in testosterone levels does not appear to be related to dopaminergic medication.^30^

Furthermore, the use of androgen deprivation therapy in patients with prostate cancer was not associated with a greater risk of PD^31,32^ (Table 1), suggesting that having low levels of androgen is not a risk factor to develop PD. The risk of developing parkinsonism at the end of the study follow-up was lower in androgen deprivation therapy users, suggesting that androgen deprivation therapy might have a slight neuroprotective effect.^32^ Thus, this suggests that androgens do not play a protective role but may actually intensify toxicity of the nigrostriatal dopaminergic pathway.

## Endogenous Androgens and Animal Models of PD

The effect of castration to reduce gonadal endogenous androgen levels was investigated on brain DA markers. Whereas castration in very young male mice increased glial activation, decreased striatal DA levels and tyrosine hydroxylase positive cells in striatum and substantia nigra, and impaired locomotor activities, this effect was age dependent, and castration in adult male mice did not induce any of these effects.^33^ Furthermore, in the 6-hydroxydopamine (6-OHDA)-lesioned rat model of PD, castration is reported to reduce 6-OHDA-induced toxicity (Table 2): castrated male rats having less DA content or neuronal loss and a decrease in motor asymmetry and oxidative stress generation following a 6-OHDA lesion.^34–36^

**Table 2.** Effect of Endogenous and Exogenous Androgen Compounds in Animal Models of Parkinson's Disease

Animal models: effect of castration	Decreased toxicity to toxin	No change in response to toxin
6-OHDA-lesioned castrated male rats	Castrated male rats having less DA content or neuronal loss after a 6-OHDA lesion^34,35^	
MPTP castrated male mice		No difference in susceptibility to MPTP is reported^37,38^

**Animal models: effect of androgen and related compound treatments**	**Active compounds**	**Inactive compounds**
Neuroprotection studies		
MPTP male mice	Dutasteride^39,40^	Testosterone^50^
	DHEA^80^	Dihydrotestosterone^50^
		Finasteride^39^
MPTP castrated male mice		Testosterone^38^
6-OHDA-lesioned gonadectomized female and male rats		Dihydrotestosterone^34,51^
Dyskinesia studies		
6-OHDA-lesioned female and male rats	Finasteride^71^	
6-OHDA-lesioned male rats	Finasteride,^72^ but impaired L-Dopa motor activation	
6-OHDA-lesioned male rats	Dutasteride^72^	

DA, dopamine; DHEA, dehydroepiandrosterone; MPTP, 1-methyl-4-phenyl-1,2,3,6-tetrahydropyridine; 6-OHDA, 6-hydroxydopamine.

Striatal DA and its metabolite dihydroxyphenylacetic acid contents in male mice were reported to be the same in intact and castrated retired breeder male mice (about 6 months old) and similarly decreased when lesioned with the neurotoxin 1-methyl-4-phenyl-1,2,3,6-tetrahydropyridine (MPTP; 4 × 10 mg/kg), a mouse model of PD.^37^ Younger 10–12 weeks old male mice showed no difference in striatal tyrosine hydroxylase staining between intact and castrated animals and a similar loss with MPTP (4 × 20 mg/kg) lesioning.^38^ The above studies had differences in species and age of animals as well toxins to model PD. Furthermore, for 6-OHDA-lesioned male rats and MPTP-treated male mice, testosterone or dihydrotestosterone treatment, the two more abundant, or more biologically active androgens, had no effect on toxin-induced lesion in castrated animals.^34,38^

Thus, in animal models, castration (reducing testosterone and dihydrotestosterone) did not increase susceptibility to toxin damaging the nigrostriatal system nor was testosterone or dihydrotestosterone treatment beneficial.

MPTP-lesioned male mice were reported to have reduced levels of plasma and brain testosterone and dihydrotestosterone compared with those of control mice.^39,40^ Leydig cells are the major site for producing endogenous testosterone under physiological conditions and a decrease in Leydig cell counts was reported in MPTP mice.^41^ This could explain the reduced plasma and brain testosterone and dihydrotestosterone levels in MPTP male mice. Activity of the steroidogenesis enzymes could also be altered by exposure to reactive oxygen species,^42,43^ which are produced after MPTP administration. In the 6-OHDA-unilaterally lesioned male rat model of PD, no difference of testosterone and dihydrotestosterone striatal and cerebral cortex was measured between the ipsilateral and contralateral sides and compared with that in intact controls; by contrast, differences in progesterone metabolism were observed.^44^

## Treatment with Androgens in PD

Although the effect of testosterone replacement therapy on motor symptoms of PD in patients with testosterone deficiency has been investigated, the studies are scarce, the number of patients included is small or the studies are case reports, and the results are not consistent (Table 1).

A double-blind placebo-controlled trial evaluated the effect of testosterone enanthate for 8 weeks on motor symptom in PD patients with low, but in the normal range, testosterone levels.^45^ Evaluation of motor function using the Unified Parkinson's Disease Rating Scale (UPDRS) has not shown any beneficial effect of testosterone treatment in PD patients.^45^ In a prospective open-labeled pilot study in 10 testosterone-deficient men with PD, the UPDRS IV scores (Fluctuations) improved at 1 month but did not show sustainable improvement at 3 months.^46^ In this study, testosterone treatment had no effect on the UPDRS (II, Activities of Daily Living; III, Motor) and the Obeso dyskinesias rating scale.^46^ A case report of an 80-year-old man with PD with testosterone deficiency described a significant improvement in resting tremor and fine motor control after testosterone administration.^47^

In a retrospective analysis of five PD patients treated with testosterone for testosterone deficiency, several PD patients described an improvement in their PD symptoms, but this was not always associated by a change in the UPDRS motor score.^27^ As noted by the authors, this improvement could be the result of a testosterone effect on mood and energy, rather than a direct effect on PD symptoms.

It should be taken into consideration that three of the four studies reported here have included PD patients with testosterone levels below the normal range. Among the nonspecific symptoms and signs associated with testosterone deficiency are decreased energy, impaired physical performance, and mobility limitation.^48,49^ Testosterone treatment in aging men with testosterone deficiency improved energy and had a modest effect on physical function.^48,49^ Thus, as testosterone therapy can act directly on symptoms and signs associated with testosterone deficiency, it is unclear whether the improvements come from restoring testosterone levels to normal levels, thus by a direct effect on symptoms of testosterone deficiency, or rather by direct effect on motor symptoms of PD. It could also be an indirect effect through the transformation of testosterone into 17β-estradiol by aromatase.

## Treatment with Androgens in Animal Models of PD

In male mice, testosterone treatment failed to induce any protective effect against MPTP toxicity^38,50^ (Table 2). However, the lack of effect of testosterone may be the result of insufficient conversion to estradiol, or the lack of beneficial effect of androgen receptor stimulation. To specifically investigate the role of androgen receptor stimulation in neuroprotection, dihydrotestosterone, which is the most potent androgen, is a more appropriate compound than testosterone since it is not aromatized to estradiol. Studies performed in MPTP-treated male mice and 6-OHDA-lesioned gonadectomized female and male rats reported no beneficial effect of dihydrotestosterone treatment,^34,50,51^ suggesting that stimulation of the androgen receptor was not effective in inducing a protective effect. Given the absence of protection with both testosterone and dihydrotestosterone, these results suggest that testosterone is not converted in the brain into estradiol in sufficient concentration to achieve neuroprotective levels.

When testosterone is administered to aged rats, there is an improvement in motor deficits, as well as an increase in DA transporters and tyrosine hydroxylase in striatum and substantia nigra of aged male rats.^52,53^ Nevertheless, these results are observed in normal aging, not in pathological conditions such as occurring in PD. In conditions where oxidative stress is present, like in reserpine-treated aged male rats, testosterone worsened the deficits in behaviors and in nigrostriatal dopaminergic system.^52^

Dihydrotestosterone can be metabolized into 3β-diol and the latter is an agonist on estrogen receptors.^54^ We previously reported reduced plasma testosterone, dihydrotestosterone, and 3β-diol in male MPTP mice.^40,55^ In men with PD, reduced 17β-estradiol and testosterone levels were reported.^56^ The reduction of gonadal androgens in PD males and MPTP mice is related to impaired Leydig cells activity. Hence, a role of 3β-diol is difficult to decipher in PD since it is a weaker estrogen receptor agonist (binding affinity of 6 nM for ERα [vs. 0.13 for 17β-estradiol] and 2 nM [vs. 0.12 for 17β-estradiol] for ERβ)^54^ than 17β-estradiol, and its levels are reduced due to decreased levels of its metabolic precursor dihydrotestosterone.

Thus, animal and clinical studies do not support that androgens may modify the risk to develop PD. The potential beneficial effect of testosterone when combined with antiparkinsonian medication to improve PD symptoms requires larger studies to draw a clear conclusion.

## Antiandrogenic Therapies in PD

Antiandrogenic therapies include drugs inhibiting the hypothalamic–pituitary–gonadal axis, including modulators of the gonadotrophic inhibitory hormone and Kisspeptin–Kiss1 receptor axis and gonadotrophic releasing hormone agonists (leuprolide, goserelin, and triptorelin) and antagonists (degarelix), androgen receptor inhibitors (cyproterone, spironolactone, eplerenone, and flutamide), and 5α-reductase inhibitors (finasteride and dutasteride). Androgen receptor inhibitors and 5α-reductase inhibitors provide prompter antiandrogenic actions and some were tested in PD patients. The major representatives of androgen receptor inhibitors are spironolactone and eplerenone, also acting on the mineralocorticoid receptor.^57,58^

In a case report, spironolactone was observed to worsen PD symptoms^59^ and no data is available for eplerenone in PD. Flutamide was investigated in rats where it was reported that low doses of flutamide reduced haloperidol-induced catalepsy and higher doses worsen catalepsy.^60^ In a dopaminergic cell line (N27 cells), flutamide inhibited testosterone-induced apoptosis,^61^ and apoptosis effect of testosterone was recently reported to be mediated by a membrane androgen receptor in N27 cells.^62^ A case report showed that low-dose cyproterone acetate treatment reduced sexual acting out in a man with PD and dementia without relevant side effects.^63^ As reviewed above, there are limited studies with the androgen receptor inhibitors in PD, whereas the 5α-reductase inhibitors have led to recent interesting findings.

### 5α-reductase

5α-reductase enzymes are enzymes that catalyze the conversion of progesterone into dihydroprogesterone and also metabolize testosterone into dihydrotestosterone. Both 5α-reductase types 1 and 2 are expressed in the brain.^64^ In the rat brain, 5α-reductase isoform 2 is localized in neurons, but not in glial cells, whereas isoform 1 is expressed in glial cells,^65,66^ suggesting different functions of these isoforms in the regulation of neuroendocrine processes. 5α-Reductase inhibitors, such as finasteride and dutasteride, are used in the clinic to treat endocrine condition such as benign prostatic hyperplasia and androgenic alopecia.^67^ Finasteride inhibits selectively 5α-reductase type 2, whereas dutasteride has higher potency than finasteride in inhibiting both types 1 and 2.^68^

### 5α-Reductase inhibitors: PD and animal models

Studies have shown a role of 5α-reductase inhibitors in dopaminergic transmission, with potential therapeutic effects in several disorders associated with dopaminergic hyperactivity.^69^ Regarding PD, a case study with two male patients with PD reports that finasteride treatment reduced pathological gambling, a side effect induced by dopaminergic medication^70^ (Table 1).

In both female and male rats lesioned with 6-OHDA, finasteride reduces the development and expression of L-Dopa-induced dyskinesias^71^; this effect is also observed with dutasteride in 6-OHDA-lesioned male rats.^72^ Lower dose of dutasteride compared to finasteride are required to produce this effect.^72^ Moreover, dutasteride does not affect L-Dopa-induced motor activation, unlike finasteride.^72^ Finasteride has been reported to attenuate behaviors induced by DA D1 and D3 receptors agonists, thus suggesting the implication of these receptors in its activity to decrease dyskinesias.^73,74^ Furthermore, both dutasteride and finasteride prevent the L-Dopa-induced upregulation of striatal DA D1-receptor-related signaling pathways and D1–D3 receptor interaction.^72^ These studies suggest that 5α-reductase inhibitors could be beneficial to reduce side effect related to dopaminergic medication such as L-Dopa-induced dyskinesias and compulsive behavior.

## Antiandrogen Therapies in Animal Models of PD

### 5α-Reductase inhibitors: neuroprotective effect

Since 5α-reductase inhibitors block the conversion of progesterone into dihydroprogesterone and also testosterone into dihydrotestosterone, and thus hypothetically increasing 17β-estradiol levels through aromatization of testosterone, these are interesting molecules as they may have a neuroprotective effect by increasing the levels of the neuroprotective steroids (Table 2).

We previously showed that administration of dutasteride to male mice starting before and pursued after MPTP lesion prevented MPTP-induced loss of DA markers, but this effect was not seen when dutasteride administration was started only after MPTP, where similar change in striatal DA content between MPTP mice and MPTP mice treated with dutasteride was observed.^39,40^ Thus, dutasteride did not increase MPTP toxicity when administration was initiated after injury. This is important information since this drug could be repurposed to reduce L-Dopa-induced dyskinesias in PD based on its decrease of abnormal involuntary movements in 6-OHDA-lesioned rats.^72^ Finasteride was ineffective in protecting dopaminergic neurons of MPTP toxicity in male mice,^39^ perhaps due to its shorter serum half-life (2 h) than dutasteride (31 h).^68^

Measures of steroid levels have shown that MPTP treatment decreased plasma and brain levels of testosterone, and dutasteride administration in MPTP mice maintains the levels of this steroid at control value, whereas the levels of dihydrotestosterone were found to be decreased in intact mice, MPTP and dutasteride-treated MPTP mice.^40^ Since testosterone or dihydrotestosterone treatments in MPTP male mice did not induce any protective effect,^50^ it seems unlikely that maintaining the physiological levels of testosterone could be one of the mechanisms by which dutasteride prevented the MPTP-induced toxicity, but rather support the protective effect of dutasteride.

Although 17β-estradiol levels were not assayed specifically in the striatum and the substantia nigra, levels of 17β-estradiol were under detection limits in the plasma and one brain hemisphere of control male mice and remained undetectable with the MPTP lesion and dutasteride treatment, suggesting that dutasteride protective effect is unlikely mediated by increasing 17β-estradiol levels.^39,40^

Although plasma and brain concentrations of progesterone are at control levels with the administration of dutasteride in intact mice, MPTP and dutasteride-treated MPTP mice have elevated progesterone levels.^40^ Thus, the protective effect of dutasteride does not seem to be only related to change in progesterone and testosterone contents and their metabolites.

Dutasteride increases dopamine transporter (DAT) specific binding and glycosylation in intact male mice, therefore, increasing DAT function at the membrane.^40^ Whereas previous study reported that mice overexpressing the DAT are more susceptible to MPTP toxicity,^75^ thus that increased DAT activity induces a detrimental effect, this is not supported by our previous study showing that the increased maturation of DAT and its activity with dutasteride treatment did not intensify MPTP toxicity, suggesting that these effects on DAT would contribute to the protection of DA neurons.^40^ Neuroprotection by dutasteride in MPTP-treated mice is also associated with reduced neuroinflammation as assessed with striatal glial fibrillary acidic protein levels, thus supporting its anti-inflammatory activity in its mechanism of action.^40^

## Androgen Precursors, Dehydroepiandrosterone and Pregnenolone for PD

Dehydroepiandrosterone (DHEA) and pregnenolone are steroids precursors in the synthetic pathways of androgens. Pregnenolone is an FDA-approved drug under investigation in clinical trials on psychiatric disorders with dysfunctions of DA signaling, including bipolar disorders, schizophrenia, and marijuana intoxication.^76^ In the 6-OHDA unilaterally lesioned male rat model of PD, DHEA levels in the striatum and cerebral cortex were unchanged by the lesion, whereas pregnenolone levels were reduced in the lesioned and unlesioned striatum but not in the cerebral cortex.^44^ Mouse brain and plasma levels of DHEA were unchanged by the MPTP lesion, whereas pregnenolone levels were reduced in the plasma and elevated in the brain by the MPTP lesion.^40^

There are limited data available on pregnenolone and DHEA in PD patients' cerebrospinal fluid, plasma, and/or brain. DHEA and its sulfate derivative were unchanged in PD patients.^77^ In animal models of PD, beneficial effects of DHEA on motor behavior were reported in MPTP parkinsonian monkeys^78,79^ and neuroprotection of dopaminergic markers against MPTP toxicity.^80^ There is potential of pregnenolone for treatment of PD and L-Dopa-induced dyskinesias. It can rescue synaptic defects and normalize hyperdopaminergic activity and abnormal DA-dependent behavior in rats offspring exposed to cannabis during pregnancy.^81^ Pregnenolone rectifies DA neuron excitability and prevents Δ9-tetrahydrocannabinol (THC)-induced enhancement of striatal DA levels. These effects were still evident when pregnenolone is cleared from the brain, indicating its long-lasting properties in counteracting pathological hyperdopaminergic states.^81^

## Discussion

Aging is the primary risk factor for PD and is associated with reduced gonadal function in both men and women. In women, the loss of ovarian function at menopause around 50 years of age is abrupt, whereas in men there is a more progressive and slower reduction of gonadal function and decrease of androgens called andropause.

Considering the sex difference in PD pointing to a protective role of ovarian steroids that is lost at menopause and the abundant literature of neuroprotective activity of estrogens and progesterone in animal models of PD, hormonal replacement (estrogen and progesterone) seems a plausible approach (reviewed in Ref.^18^). However, the risk associated with estrogens has led to search for alternatives such as selective estrogen receptor modulators, raloxifene, specific agonists for estrogen receptor subtypes (estrogen receptor α, estrogen receptor β, and membrane estrogen receptor GPER1).^18^ As reviewed above, androgen loss (due to aging or castration) and androgen treatment have not given solid beneficial or deleterious evidence in PD.

Although this review has mainly focused on androgens in men and PD, androgen variations throughout life are also reported in women. Serum androgen levels in women decline in the early reproductive years but levels do not decline further with the menopause transition.^82^ The decline of testosterone levels in women is of 55%.^82^ The higher androgens relative to estrogen in women in the postmenopausal state and its role in the increase in PD incidence after menopause remain to be investigated. Nevertheless, in gonadectomized female rats, dihydrotestosterone treatment has no effect on 6-OHDA toxicity, whereas estradiol showed protective effect,^34^ suggesting that increasing androgen levels in females have no damaging effect in the dopaminergic system.

Many cellular mechanisms contributing to impaired neuronal function during aging are also present in PD, including mitochondrial dysfunction, inflammation, oxidative stress, and impaired DA metabolism.^83–85^ More specifically for brain DA in aging, there is a decrease in the synthesis of DA, DA receptors and transporters, as well as tyrosine hydroxylase positive neurons.^83,85–87^ The age-related decrease in brain DA activity is associated with a decline in cognitive and motor functions for both men and women.^88–90^ Changes during aging could render DA neurons more vulnerable to insults. Indeed, the toxin MPTP produces greater degeneration of DA neurons in aged monkeys and mice than in younger animals.^91,92^

Most people will age without developing PD. What causes the degeneration of DA neurons in PD is still unknown and is likely a multifactorial etiology including genetic and environmental factors.^84^ The vulnerability of DA neurons observed with aging could reduce the ability of those neurons to respond to stressful events, and a therapeutic strategy that targets the multiple mechanisms contributing to DA neuron dysfunction should be useful.

Although the loss of DA nigrostriatal neurons is the major neuropathological cause of PD, other neuronal groups also degenerate to a lesser extent such as serotoninergic neurons of the raphe nucleus, noradrenergic neurons of the locus coeruleus, or cholinergic neurons of the nucleus basalis of Meynert.^93^ By contrast, brain glutamate neurotransmission is reported to be increased in PD.^94^ PD also involves accumulation of intracellular α-synuclein protein deposits called Lewy bodies.^95^ Endocrine drugs with multiple activities could have translational value for PD. Among these activities are the anti-inflammatory action of various steroids that could be useful for PD. Indeed, neurodegenerative diseases including PD are associated with inflammation.^96^

Viral infections were proposed as potential risk factors for PD, and there is supporting although not entirely consistent epidemiological and basic science supporting evidence (review Ref.^97^). In a multihit hypothesis of PD, Sadasivan et al. demonstrated that prior exposure of mice to non-neurotropic pandemic influenza A/California/04/2009 H1N1 virus, which triggers brain inflammation, exacerbates their vulnerability to a parkinsonian toxin, MPTP, 1 month later, resulting in heightened loss of DA neurons.^98^ This finding raises a concern for survivors of viral infections, who could be more susceptible to other potential environmental PD triggers, which independently are not considered sufficient to elicit PD phenotypes.

Since men have been shown to be over-represented among those severely affected by coronavirus disease (COVID-19), repurposing drugs for COVID-19 with an endocrine perspective has been recently reviewed.^99^ Interestingly, 5-α reductase inhibitors (finasteride and dutasteride) were recently shown to have beneficial effects in males with COVID-19.^99–102^ Therefore, with the possible increase of parkinsonism post-COVID-19 infection and the higher incidence of men in both these diseases, possible converging endocrine treatments open interesting opportunities for drug repurposing.

## Conclusion

Although it is now well documented that PD is more prevalent in men than in women, androgens have not consistently shown beneficial or a deleterious effect on PD symptoms or disease progression. Numerous antiandrogen drugs are available to treat endocrine conditions, thus offering opportunities to repurpose them for PD. The 5α-reductase inhibitors have shown neuroprotective and antidyskinetic activities and need to be further investigated. Although the effect of dutasteride was observed only when started before injury, the lack of increased damage to dopaminergic neurons when used after the lesion makes it an attractive drug for repurposing in PD patients for its antidyskinetic properties.^72^

Moreover, although testosterone derivatives and related compounds (such as anabolic-androgenic steroids) are frequently misused by athletes, they offer possibilities that could be helpful in PD neurodegeneration condition (reviewed Ref.^103^). Selective androgen receptor modulators (SARMs) are compounds developed to be tissue-selective androgen receptor ligands.^103,104^ SARMs give an alternative for androgens therapy (osteoporosis, prostate cancer, and muscle wasting), but are presently recognized as forbidden substances by the World Anti-Doping Agency.^104^ Flutamide, initially classified as an androgen receptor inhibitor, is now considered as an SARM. The activity of SARMs in the normal brain and in PD brain is yet to be investigated.

## Abbreviations Used

6-OHDA: 6-hydroxydopamine
COVID-19: coronavirus disease
DA: dopamine
DAT: dopamine transporter
DHEA: dehydroepiandrosterone
MPTP: 1-methyl-4-phenyl-1,2,3,6-tetrahydropyridine
PD: Parkinson's disease
SARM: selective androgen receptor modulator
UPDRS: Unified Parkinson's Disease Rating Scale
